# Gas-Phase and Surface-Initiated Reactions of Household Bleach and Terpene-Containing Cleaning Products Yield Chlorination and Oxidation Products Adsorbed onto Indoor Relevant Surfaces

**DOI:** 10.1021/acs.est.3c06656

**Published:** 2023-11-27

**Authors:** Cholaphan Deeleepojananan, Vicki H. Grassian

**Affiliations:** Department of Chemistry and Biochemistry, University of California San Diego, La Jolla, California 92093, United States

**Keywords:** indoor surface chemistry, cleaning products, silica, rutile, hypochlorous acid, limonene

## Abstract

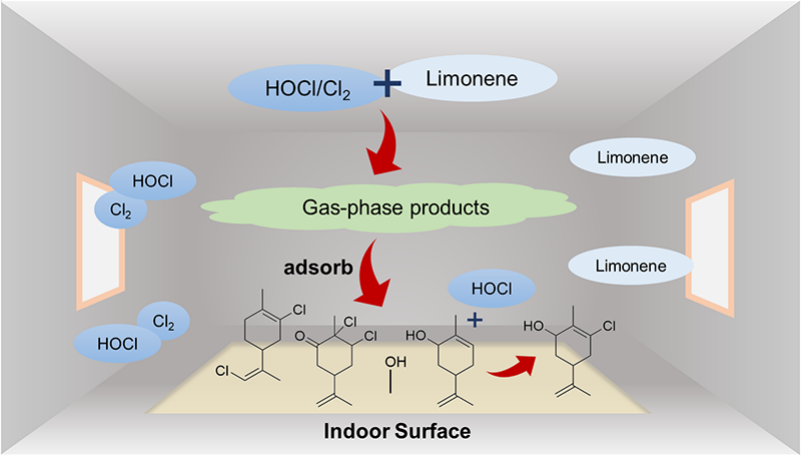

The use of household bleach cleaning products results in emissions of highly oxidative gaseous species, such as hypochlorous acid (HOCl) and chlorine (Cl_2_). These species readily react with volatile organic compounds (VOCs), such as limonene, one of the most abundant compounds found in indoor enviroments. In this study, reactions of HOCl/Cl_2_ with limonene in the gas phase and on indoor relevant surfaces were investigated. Using an environmental Teflon chamber, we show that silica (SiO_2_), a proxy for window glass, and rutile (TiO_2_), a component of paint and self-cleaning surfaces, act as a reservoir for adsorption of gas-phase products formed between HOCl/Cl_2_ and limonene. Furthermore, high-resolution mass spectrometry (HRMS) shows that the gas-phase reaction products of HOCl/Cl_2_ and limonene readily adsorb on both SiO_2_ and TiO_2_. Surface-mediated reactions can also occur, leading to the formation of new chlorine- and oxygen-containing products. Transmission Fourier-transform infrared (FTIR) spectroscopy of adsorption and desorption of bleach and terpene oxidation products indicates that these chlorine- and oxygen-containing products strongly adsorb on both SiO_2_ and TiO_2_ surfaces for days, providing potential sources of human exposure and sinks for additional heterogeneous reactions.

## Abstract 2

The use of oxidative cleaning agents and the presence of limonene lead to subsequent chemical reactions and the formation of gas-phase products indoors. This study investigates the interactions of these products and their chemical transformations on indoor relevant surfaces.

## Introduction

Humans spend approximately 90% of their time indoors while undertaking various activities, such as cooking, cleaning, working, and smoking.^1,2^ In particular, US adults dedicate around one hour a day cleaning their homes with different methods that accompany cleaning products; vacuuming, sweeping, wiping, and mopping.^3−5^ The use of chemical cleaning agents can release a wide range of volatile species indoors.^4,6−8^ Application of household cleaning and consumer products containing chlorine bleach and terpenes primarily generates gaseous species that are oxidizing agents and volatile organic compounds (VOCs), respectively, enabling secondary chemical reactions within indoor environments.^9−12^ Reactions of prevalent indoor monoterpenes (C_10_H_16_), namely, limonene and alpha-pinene, with indoor oxidants such as hydroxyl radicals (OH), nitrate radicals (NO_3_), and ozone (O_3_) have been extensively studied.^13−18^ Most of these reactions lead to the formation of secondary organic aerosols (SOAs), which are indoor air pollutants and potential respiratory and pulmonary irritants.^19−21^ Specifically, limonene is one of the most abundant indoor VOCs commonly found in fragranced cleaning products and room deodorizers due to its lemon scent with an average indoor concentration of 5–15 ppb^18^ and a reported maximum concentration of hundreds of ppb during product use.^22^ Limonene can also undergo gas-phase reactions with hypochlorous acid (HOCl) and chlorine (Cl_2_) that are emitted from household bleach cleaning products.^11,12^ Sodium hypochlorite (NaOCl) is an active ingredient in aqueous bleach solutions (average pH 12).^23^ Consequently, HOCl and Cl_2_ are produced and released into the indoor environment as bleach solutions are acidified as a result of carbon dioxide uptake.^24^ Indoor concentrations of HOCl and Cl_2_ have been seen to reach a maximum of 370 and 130 ppbv, respectively, during mopping events in a test house.^10^ Interestingly, both HOCl and Cl_2_ decay faster than the expected air exchange rate, owing to their partitioning onto indoor surfaces.^10,24^ HOCl is known to readily react with unsaturated organic compounds containing carbon–carbon double bonds to form chlorohydrins, whereas Cl_2_ can also react with unsaturated molecules by Cl additions across the double bond (see Supporting Information (SI) Scheme S1).^25−27^ Limonene gets quickly oxidized by HOCl and Cl_2_ in the gas phase due to having both endo- and exocyclic double bonds in its structure.^11,12^

In addition to outdoor air exchange and gas-phase chemical reactions, a large amount of indoor gas-phase species is removed through surface partitioning.^28^ Examples of ubiquitous indoor relevant surfaces include window glass, paintings, wood, and wallboards. The indoor surface-to-volume ratio is approximately by 300 times larger than that of the outdoors.^26^ Therefore, surfaces play a unique role in indoor environments by serving as a reservoir or a sink for adsorption of gas-phase species,^29−31^ particle deposition,^32,33^ organic film growth,^34−36^ and heterogeneous reactions.^25,37,38^ Moreover, compounds that interact and/or react on surfaces can be released back into the indoor air.^39^ This can impact indoor air quality on longer time scales. Hence, studies of chemical transformations on indoor relevant surfaces are essential inputs for indoor air quality modeling.^26^

In this work, we utilized high surface area silica (SiO_2_) as a proxy for window glass and rutile (TiO_2_) as a component of paint and self-cleaning surfaces, to study surface transformations during exposure to indoor prevalent gaseous species generated from common household cleaning products, chlorine bleach, and limonene-containing products. Different oxidized and chlorinated VOCs are characterized by these reactions, and we propose possible reaction mechanisms for their formation.

## Materials and Methods

### Environmental Teflon Chamber Experiment

A 240-L environmental Teflon chamber made of fluorinate ethylene propylene (FEP) film (American Durafilm, MA) was used to simulate indoor environments. The schematic of the chamber was previously reported elsewhere.^40^ Prior to exposure, the chamber was flushed with a high flow of zero air overnight to prevent contamination and excessive moisture (RH < 15%, typically 10–12%, as measured by a digital humidity sensor (Sensirion SHT85) placed at the chamber outlet). The chamber inlet was connected to a glass mixing chamber, which was cleaned and sonicated with methanol (HPLC grade, Fisher Scientific) and water prior to use. All other glassware and vials used in these experiments were calcined at 500 °C to remove trace organics, and all aqueous solutions were prepared using Milli-Q water (18.2 MΩ cm). All experimental conditions were optimized to overcome the limit of detection of analytical methods used (UV–vis spectroscopy and gas chromatography–mass spectrometry (GC-MS)) so that the signals were distinguished from the background noise within the linear range of the assay. Gaseous HOCl and Cl_2_ were generated by bubbling 50 SCCM of zero air through a 0.36 M aqueous solution of NaOCl (10–15% available chlorine, Sigma-Aldrich) with a pH value adjusted to ∼6.5 using NaH_2_PO_4_·H_2_O (>98%, Acros Organics) in order to maximize HOCl production (p*K*_a, HOCl_ = 7.25 at 25 °C).^22^ It should be noted that the formation of Cl_2_ gas is unavoidable due to the slightly acidic precursor pH and Cl^–^/Cl_2_ equilibria.^25^ The gaseous HOCl and Cl_2_ produced were passed through a HEPA filter (HEPA-Vent 50 mm disc, Whatman) to remove aerosols before entering the glass mixing chamber. Limonene vapor was produced by flowing 0.5 SLPM of zero air into a two-necked round-bottom flask containing 250 μL of liquid (+)-limonene (>99.0%, TCI America). Additionally, 3 SLPM of zero air was introduced to the mixing chamber as a carrier gas. Thorough mixing of all gaseous species was ensured by using a magnetic stir bar at a constant speed before introducing into the blank Teflon chamber for approximately 1 h. All tubing material used in these experiments was Teflon except the minimal amount of Tygon tubing used for the glassware ports. Prior to all experiments, SiO_2_ (Aerosil OX50, Evonik, BET surface area = 40 ± 1 m^2^ g^–1^) and TiO_2_ particles (99.9% rutile, US Research Nanomaterials, BET surface area = 17 ± 1 m^2^ g^–1^) were heated at 200 °C in an oven to remove adsorbed water and trace organic contaminants as much as possible. Thin films of indoor relevant surfaces were prepared by sonicating a 75.0 mg/mL slurry of either SiO_2_ or TiO_2_ in ethanol (200 proof, Koptec) for 20 min, and then transferring 2.00 mL of the slurry to a PTFE dish (2.48 in. diameter, Fisher Scientific) uniformly. All samples were allowed to air-dry and were placed inside the Teflon chamber at its center position. The prepared thin films were then exposed to a mixture of gaseous HOCl/Cl_2_ and limonene for 2 h. Gas-phase reaction products were collected after a 2-h exposure using an impinger containing 10.0 mL of 1:1 methanol/water solution. The impinger inlet was connected to the outlet of the Teflon chamber, whereas the other side was connected to a vacuum. Thin films were then removed from the chamber, extracted with 1.50 mL of 1:1 methanol/water, sonicated for 1 h, and centrifuged to collect supernatants for HRMS analysis. All samples were stored at −20 °C and analyzed within 24 h of collection.

HOCl/Cl_2_ and limonene concentrations were measured separately by performing two blank experiments to avoid cross-contamination in analysis. HOCl and Cl_2_ concentrations from the source were quantified by UV–vis spectroscopy with a pulsed Xe lamp and a diode array detector (Ocean Optics USB 3000) using the maximum wavelengths at 240 and 330 nm, respectively.^12,25^ The concentrations of HOCl and Cl_2_ inside the Teflon chamber were calculated based on total flow rates and the known chamber volume (240 L). For limonene quantification, an impinger was used similar to the previous description. The limonene concentration was determined by GC-MS (Thermo Trace 1300/TSQ 8000 Evo Triple Quadrupole) using external standards (Figure S1).

### High-Resolution Mass Spectrometry

The extracted gas-phase and surface products after HOCl/Cl_2_ and limonene exposure were analyzed using high-resolution mass spectrometry (HRMS, Thermo Orbitrap Elite Hybrid Linear Ion Trap-Orbitrap MS). All samples were analyzed in positive-ion mode. The heated electrospray ionization (HESI) source was operated at 100 °C. The ESI capillary was set to a voltage of 3.50 kV at 350 °C. Mass spectra were acquired with a mass range of 50–500 Da. Chemical formulas were assigned with a mass tolerance of <3 ppm with the following element ranges: ^12^C, 0–10; ^1^H, 0–30; ^16^O, 0–10; ^23^Na, 0–1; ^35^Cl, 0–2; ^37^Cl, 0–2. Other peaks with a higher mass tolerance were not considered in this work.

### Transmission-FTIR Experiment

A complementary experiment to the environmental Teflon chamber experiment was performed using transmission Fourier transform infrared (Model Nicolet iS50 FTIR, Thermo Fisher Scientific) spectroscopy. A custom-made moveable Teflon-coated infrared cell (177 ± 2 mL) was connected to a glass mixing chamber (1329 ± 2 mL) with multiple valves for gas injection, two absolute pressure transducers (MKS Instruments, Inc., 10 and 1000 Torr), and a two-stage vacuum system, including a turbomolecular pump (Agilent TwisTorr 74 FS) and a mechanical pump (Adixen Pascal 2010 SD). More details of this FTIR setup have been previously reported.^31^ Briefly, approximately 12 mg of SiO_2_ (or 20 mg TiO_2_) particles were pressed onto half of a tungsten grid (Alfa Aesar, tungsten gauze, 100 mesh woven from 0.0509 mm diameter wire) and installed into the IR cell such that the infrared beam can interchangeably pass through both the pressed sample and the bare grid in order to collect the surface and gas-phase spectra, respectively. After overnight evacuation, the sample was first exposed to limonene for 30 min at an equilibrium pressure of 77 ± 2 mTorr. Then, all gas-phase limonene in the mixing chamber was evacuated. Limonene and HOCl/Cl_2_ were not injected at the same time to avoid back pressure and contamination. Gas-phase HOCl/Cl_2_ was obtained in a 1.5-L gas bulb by flowing 100 SCCM of zero air through a bubbler containing NaOCl precursor. HOCl/Cl_2_ was then introduced into the IR cell to allow for reactions with the surface-adsorbed limonene and the remaining gas-phase limonene to achieve an equilibrium pressure of 87.1 Torr. Note that zero air was also included in the bulb and the new equilibrium pressure was reached within seconds. The IR cell was isolated after one min of HOCl/Cl_2_ injection. The equilibrium partial pressures of HOCl and Cl_2_ inside the IR cell were approximately 21 and 48 mTorr, respectively. The reaction was allowed to proceed for another 2 h for equilibration, and then the entire system was evacuated for 1 h. Single-beam spectra of the surface and the gas phase were collected with 250 scans at a resolution of 4 cm^–1^ over the spectral range extending from 650 to 4000 cm^–1^. The resulting FTIR spectra of the surface upon adsorption and after evacuation were obtained by reprocessing with their corresponding background single-beam spectra and subtracting the corresponding reprocessed gas-phase spectra.

### Reaction Thermodynamics Calculations

The Gibbs free energy (Δ*G*°) values of all proposed reaction pathways were calculated using Spartan’20 Software (Wavefunction Inc.) at the B3LYP/6-311+G** level of theory to confirm the thermodynamics of the reactions (in the gas phase). The molecular mechanics energy of all molecules was minimized based on the Merck Molecular Force Field.

## Results and Discussion

### High-Resolution Mass Spectra of Gas-Phase and Surface Products

**Figure 1:**
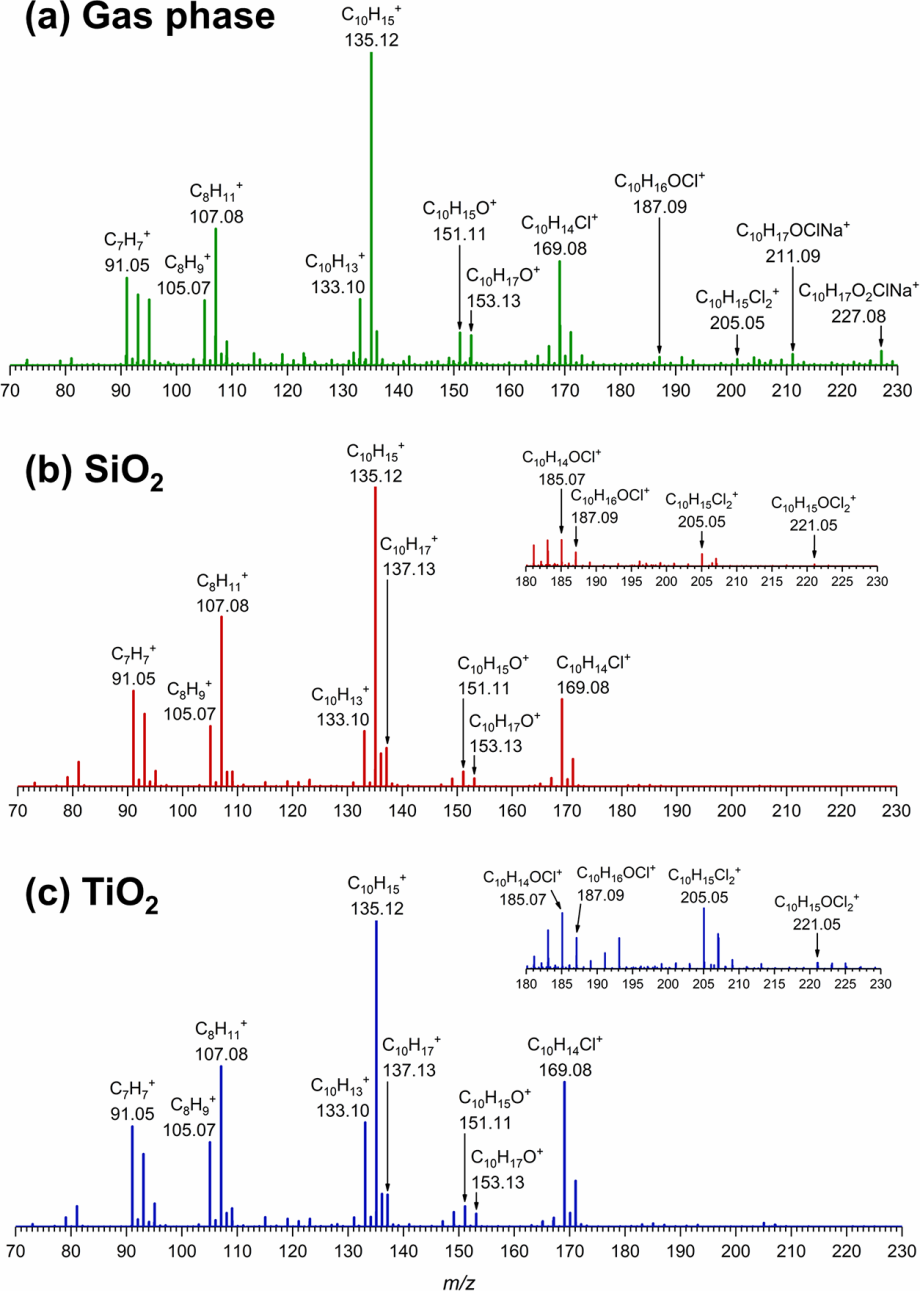
Normalized mass spectra in positive-ion mode of (a) gas-phase reaction products and surface products extracted from (b) SiO_2_, and (c) TiO_2_ after exposure to HOCl/Cl_2_ and limonene for 2 h in the Teflon chamber. The inset in (b) and (c) show an expanded view of higher m/z.

Following the environmental Teflon chamber experiments, the equilibrium mixing ratios of HOCl, Cl_2_, and limonene present in the chamber were determined to be approximately 1.7, 2.2, and 12 ppm, respectively. The partial pressures of HOCl, Cl_2_, and limonene in zero air were calculated to be approximately 1.3, 1.7, and 9.1 mTorr, respectively. Tandem mass spectrometry (MS/MS) was further used to identify fragment ions of a molecular ion and to further investigate the structure of each product formed. The mass spectrum of gas-phase reaction products between HOCl/Cl_2_ and limonene (Figure 1a) revealed a parent ion of limonene with an addition of O and Cl at *m*/*z* 187.09, which was attributed to C_10_H_16_OCl^+^ with prominent fragment ions confirmed by MS/MS, including *m*/*z* 169.08 (C_10_H_14_Cl^+^), 151.11 (C_10_H_15_O^+^), 133.10 (C_10_H_13_^+^), 105.07 (C_8_H_9_^+^), and 91.05 (C_7_H_7_^+^). These mass-to-charge ratios are similar to the mass spectral data reported in the study of bleach and limonene dark reactions in the gas phase by Wang et al.^12^ Additionally, a limonene oxidation product was detected at *m*/*z* 153.13 (C_10_H_17_O^+^) with the following fragment ions: *m*/*z* 151.11 (C_10_H_15_O^+^), 135.12 (C_10_H_15_^+^), and 107.08 (C_8_H_11_^+^). The chemical structure of this limonene oxidation product at *m*/*z* 153.13 was confirmed by comparing with mass spectra of standard compounds (1000 ppm in methanol), including carvone (C_10_H_14_O), carveol (C_10_H_16_O), alpha-terpineol (C_10_H_18_O), and terpinen-4-ol (C_10_H_18_O) (Figure S2a–d). It was identified that the mass spectrum of carveol matched that of the *m*/*z* 153.13 compound. Furthermore, we observed compounds with addition of two or more atoms of either O or Cl to the limonene backbone, namely, *m*/*z* 205.05 (C_10_H_15_Cl_2_^+^), 211.09 (C_10_H_17_OClNa^+^), and 227.08 (C_10_H_17_O_2_ClNa^+^). The presence of these compounds indicates that reactions of limonene with more than one HOCl/Cl_2_ molecule may occur. A list of compounds detected by HRMS along with their fragment ions is summarized in Table 1. The molecular ions containing the Cl isotope, ^37^Cl, were also observed, which verified the assignments of the Cl-containing compounds (Table S1).

**Table 1:**
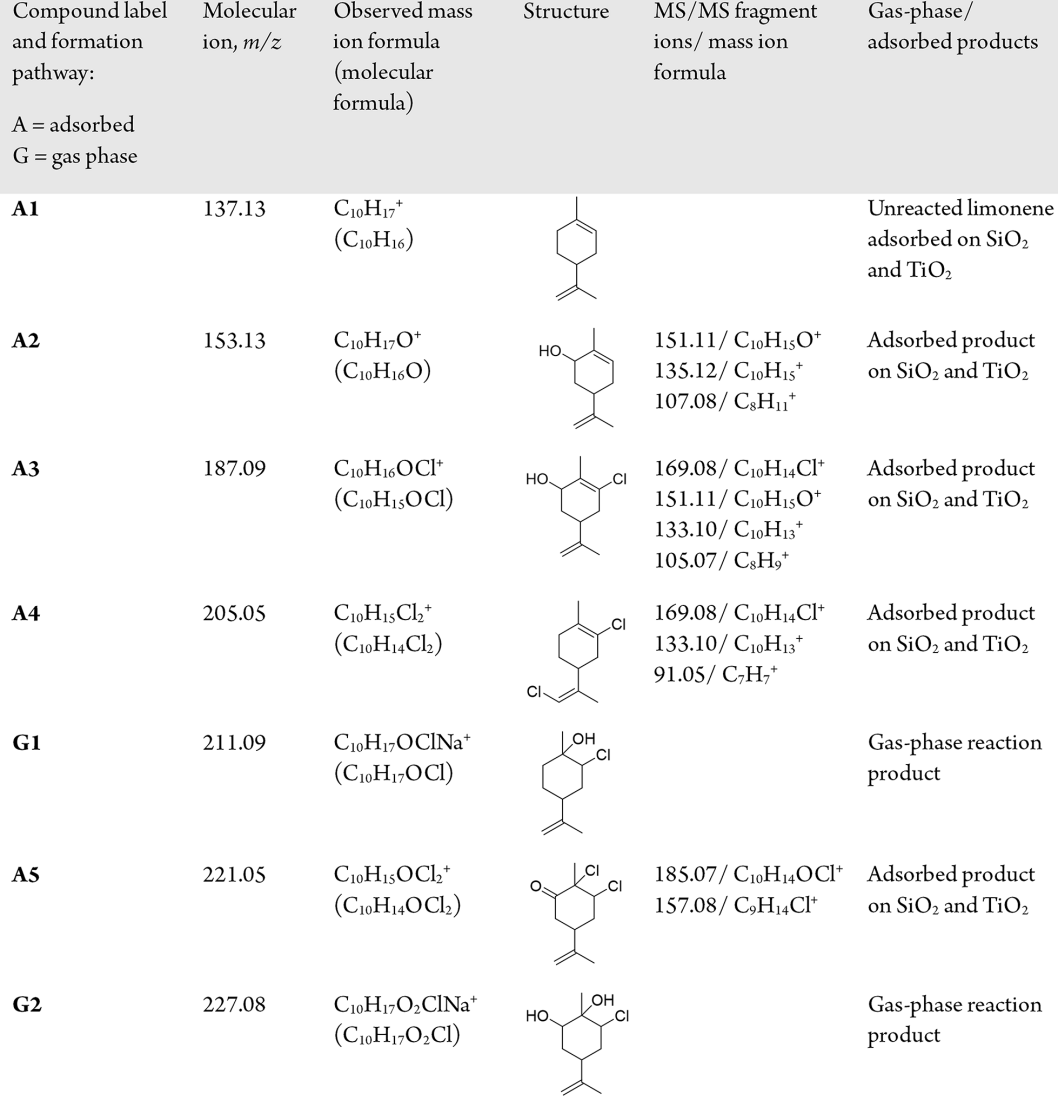
List of Assigned Compounds and Fragments Obtained by HRMS from the Extracted Gas-Phase and Surface Products Following the Exposure of SiO_2_ and TiO_2_ Surfaces to Limonene and HOCl/Cl_2_

Interestingly, the mass spectra of the surface products extracted from SiO_2_ (Figure 1b) and TiO_2_ (Figure 1c) exhibited a pattern similar to that of the gas-phase reaction products, including *m*/*z* 153.13 (C_10_H_17_O^+^), 187.09 (C_10_H_16_OCl^+^), 205.05 (C_10_H_15_Cl_2_^+^), 221.05 (C_10_H_15_OCl_2_^+^), and their fragment ions. Therefore, SiO_2_ and TiO_2_ surface products are most likely due to the adsorption of the gas-phase reaction products. Additionally, a minor peak of unreacted limonene (C_10_H_17_^+^) was also observed at *m*/*z* 137.13. Proposed formation pathways are discussed (*vide infra*). All gas-phase and surface products observed in HRMS are summarized in Table 1 along with their origin, i.e., whether the compounds are from the parent VOC (limonene) or the adsorbed gas-phase products.

### Transmission-FTIR Spectra of Surface Products

**Figure 2:**
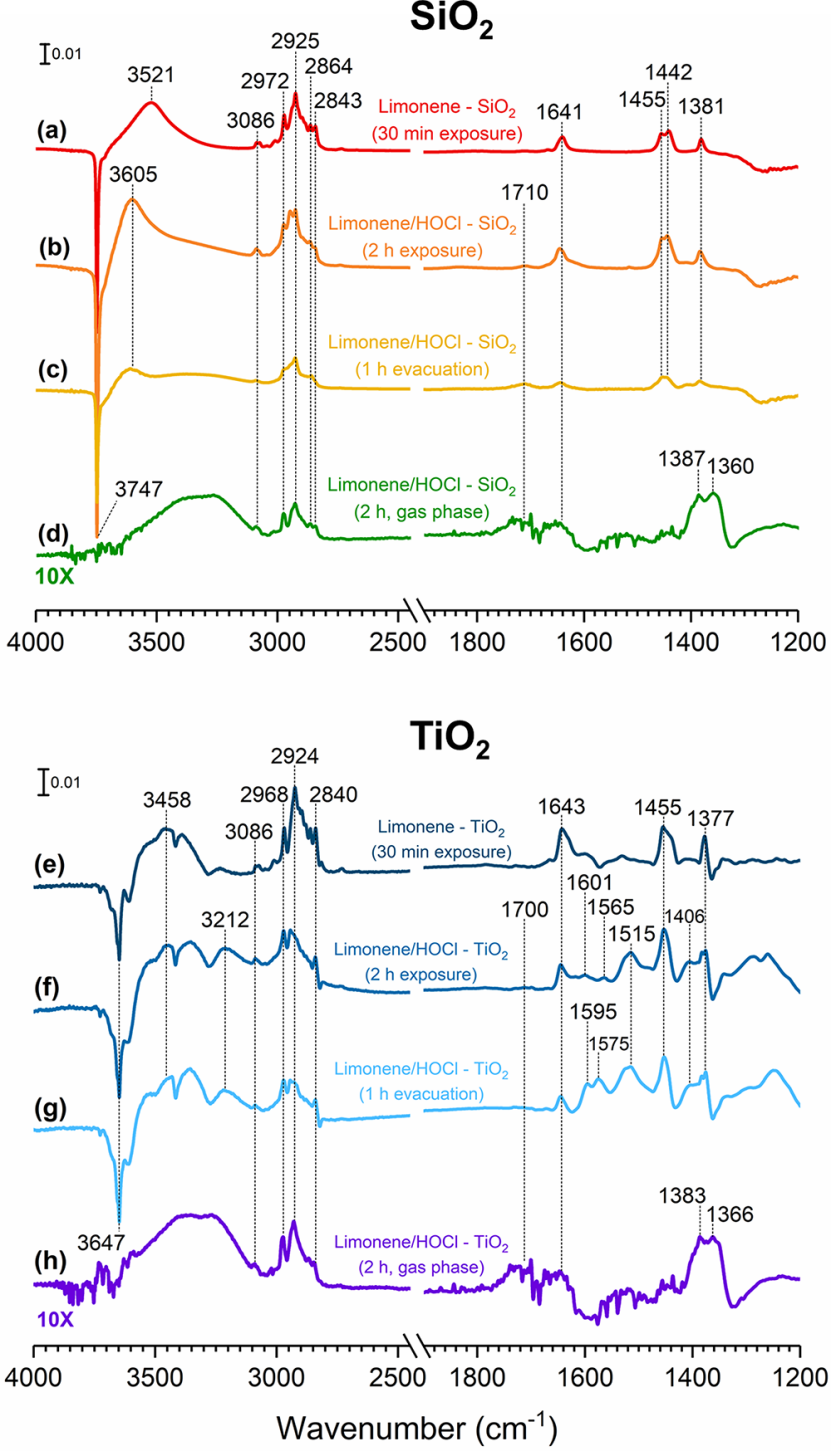
Top panel: FTIR spectra of SiO_2_ after exposure to (a) limonene for 30 min followed by (b) HOCl/Cl_2_ for 2 h and (c) 1 h of evacuation. (d) The gas-phase spectrum in the presence of SiO_2_ was collected at 2 h of exposure to limonene and HOCl/Cl_2_. Bottom panel: FTIR spectra of TiO_2_ after exposure to (e) limonene for 30 min followed by (f) HOCl/Cl_2_ for 2 h and (g) after 1 h of evacuation. (h) The gas-phase spectrum in the presence of TiO_2_ was also collected at 2 h of exposure to limonene and HOCl/Cl_2_.

FTIR spectra of SiO_2_ and TiO_2_ surfaces after exposure to limonene and HOCl/Cl_2_ followed by a 1-h evacuation collected at room temperature under dry conditions (RH < 10%) are shown in Figure 2. Adsorption of limonene and HOCl/Cl_2_ reaction products on the SiO_2_ surface revealed a few spectral changes compared to the vibrational frequencies of pure limonene exposed to a hydroxylated SiO_2_ surface (Figure 2a). Previous studies by our group had shown that limonene reversibly adsorbed on the SiO_2_ surface through π-hydrogen bonding,^31,41^ resulting in the loss of silanol groups at 3747 cm^–1^ and a red-shifted broad band appearing around 3500 cm^–1^. Following HOCl/Cl_2_ exposure (Figure 2b), a negative sharp peak at 3747 cm^–1^ corresponding to the loss of isolated hydroxyl groups on the SiO_2_ surface and a red-shifted broad band centered at 3600 cm^–1^ were observed. This broad absorption band was centered at approximately 100 cm^–1^ higher than the band from the limonene-SiO_2_ FTIR spectrum (Figure 2a), which was around 3500 cm^–1^, indicating that different hydrogen bonding interactions between the SiO_2_ surface and the limonene/HOCl products may occur. Moreover, these spectral features still remained on the surface even after 1 h of evacuation (Figure 2c).

According to the HRMS results showing the presence of oxygenated and chlorinated compounds from SiO_2_ surface extraction (Figure 1), it is possible that the H atom of the surface hydroxyl groups interacted with these oxygenated and chlorinated limonene molecules through hydrogen bonding with either the O or Cl atoms. Such interaction results in a different shift in vibrational frequencies than the π-hydrogen bonding shift found for limonene. Adsorption of oxygenated terpenes, such as carvone and alpha-terpineol on SiO_2_, were also previously studied using FTIR spectroscopy.^42,43^ These compounds also interact with SiO_2_ surfaces primarily *via* hydrogen bonding, resulting in slower desorption kinetic rates for these products.

In this study, the C–H stretching vibrations of the sp^3^ carbon were present from *ca.* 2840–2975 cm^–1^ and the sp^2^ carbon from the vinyl group was observed at 3086 cm^–1^. Other C–H bending modes were also present at around 1381, 1442, and 1455 cm^–1^. Moreover, a small C=O stretch was observed at 1710 cm^–1^, suggesting that the adsorption of compounds containing carbonyl groups is possible. Additionally, a band at 1641 cm^–1^ due to the C=C stretching mode became apparent in the spectrum. The corresponding gas-phase spectrum collected after 2 h of limonene and HOCl/Cl_2_ exposure also revealed the C–H stretching and bending modes, the C=O stretching mode, and the C=C stretching mode (Figure 2d).

A recent study has shown that limonene adsorbs on hydroxylated TiO_2_ via π-hydrogen bonding with the Ti–OH groups on the surface similarly to that of the interactions between limonene and silanol groups of SiO_2_.^44^ Such interactions were observed in this study following the exposure of TiO_2_ to limonene (Figure 2e) with a negative sharp peak at 3647 cm^–1^ and the appearance of a broad band around 3400 cm^–1^. Other vibrational modes, including C–H stretching, C–H bending, and C=C stretching modes, were also observed. The infrared spectrum for TiO_2_ is somewhat more complicated than that for SiO_2_ after 2 h of exposure to limonene and HOCl/Cl_2_ (Figure 2f). However, there are certain similarities in spectral features to those of the SiO_2_, including the C–H stretching modes of sp^2^ and sp^3^ carbons in the spectral region from *ca.* 2840–3090 cm^–1^, the C–H bending modes from 1377 to 1455 cm^–1^, the C=C stretching mode at 1643 cm^–1^, and the C=O stretching mode at 1700 cm^–1^. Rutile has been shown to exhibit interactions with organic compounds, such as carvone, benzene, and chlorobenzene on two different surface sites: isolated hydroxyl groups and surface Ti^4+^ ions.^44−46^ Therefore, given the variety of products formed on the TiO_2_ surface, they can undergo various types of interactions with either the Ti–OH or Ti^4+^ surface sites. In particular, the loss of the 3647 cm^–1^ peak due to hydrogen bonding between the surface isolated hydroxyl groups and the adsorbed products was concomitant with the positive broadband feature at around 3400 cm^–1^, suggesting a π–hydrogen bonding interaction between the Ti–OH groups and the double bonds of limonene molecules. Following exposure of TiO_2_ to limonene and HOCl/Cl_2_ for 2 h, a multiband feature from 3200 to 3400 cm^–1^ is observed, suggesting various types of hydrogen bonding interactions. Interestingly, there are additional spectral features in the spectral range from 1500 to 1600 cm^–1^, which can be a result from the adsorption of different reaction products. The corresponding gas-phase spectrum in the presence of TiO_2_ (Figure 2h) also showed the C–H stretching and bending modes, the C=O stretching mode, and the C=C stretching mode similar to the gas-phase spectrum in the presence of SiO_2_ as discussed (see Figure 2d). Notably, these surface-bound species remain strongly adsorbed on the TiO_2_ surface after 1 h of evacuation (Figure 2g) as shown by minimal spectral changes compared to the spectrum acquired prior to evacuation. Moreover, the bands at 1575 and 1595 cm^–1^ grew in intensity after evacuation, suggesting products of a surface-initiated process. Spectral assignments of other vibrational modes can be found in Table 2.

**Table 2:** Vibrational Bands Observed for Different Functional Groups on SiO_2_ and TiO_2_ after Exposure to Limonene HOCl/Cl_2_

	Experimental vibrational frequencies (cm^–1^)		
Mode	SiO_2_	TiO_2_	Literature values of vibrational frequencies (cm^–1^)
ν(MO–H, isolated)	3747	3647	3742 (SiO_2_),^31,41^ 3656 (TiO_2_)^44^
ν(O–H)	3500, 3600	3200–3400	
ν_s_(C–H, sp^2^)	3086	3086	3074^31^
ν(C–H, sp^3^)	2843–2972	2840–2968	2834–2967^31,49^
ν(C=O)	1710	1700	
ν(C=C, alkene)	1641	1643	1645^31,50^
δ(CH_2_, CH_3_)	1360–1455	1366–1455	1377, 1453^50^

In order to further understand the nature of the surface adsorbed products and to compare the results obtained in the *in situ* infrared experiments to those from the Teflon chamber experiments, the SiO_2_ and TiO_2_ samples were removed from the IR cell after 1 h of evacuation. Then, the products were extracted from the sample using a 1:1 solution of MeOH and water in the same manner previously described. The aliquots obtained after sonication and centrifugation were analyzed by HRMS. The surface-exposed equilibrium partial pressures of limonene, HOCl, and Cl_2_ for these FTIR experiments were approximately 10 times higher than those in the Teflon chamber due to the analytical limitations of the techniques. The mass spectra of the extracted SiO_2_ and TiO_2_ samples are shown in Figure S3. All of the parent and fragment ions detected were in good agreement with the previous mass spectra from the Teflon chamber experiments, confirming the formation of surface products, as summarized in Table 1.

**Figure 3:**
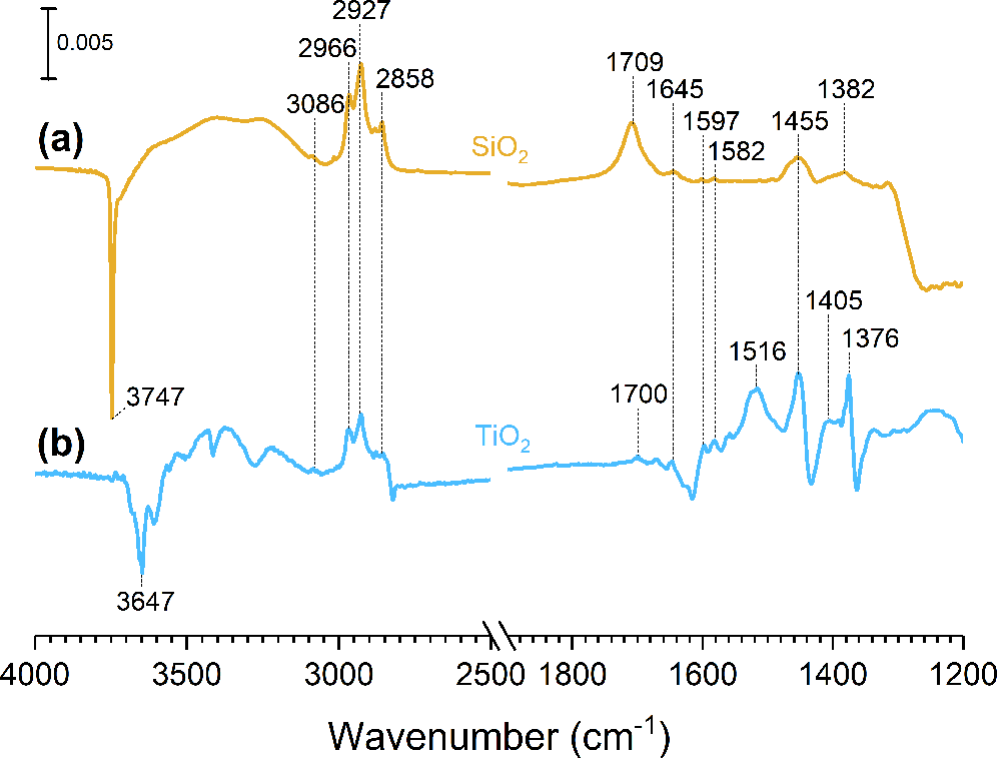
FTIR spectra of (a) SiO_2_ and (b) TiO_2_ after 2 h exposure to limonene and HOCl/Cl_2_ followed by overnight evacuation.

Furthermore, to determine the adsorption efficiency of the resulting surface products following exposure to limonene and HOCl/Cl_2_, two additional experiments were carried out. First, SiO_2_ and TiO_2_ surfaces were exposed to limonene, HOCl, and Cl_2_ for 2 h at equilibrium partial pressures of 43 mTorr, 5 mTorr, and 10 mTorr, respectively, then evacuated overnight (>20 h). The FTIR spectra of both SiO_2_ and TiO_2_ surfaces (Figure 3) showed that the surface products still remained adsorbed, suggesting a significantly slow desorption process. The hydrogen bonding and metal cation interactions between the reaction products and surfaces, in part, may account for the reduced desorption rates. In addition, small adsorbate molecules can get trapped in the interparticle pores of metal oxide surfaces,^47,48^ potentially leading to the slow desorption of these chlorinated and oxygenated compounds as well.

The mass spectra of the gas-phase reaction products and the surface products extracted from the SiO_2_ and TiO_2_ surfaces from the Teflon chamber experiments previously suggested that the formation of surface products resulted from the adsorption of the gas-phase reaction products between limonene and HOCl/Cl_2_. To determine if surface-initiated reactions could occur without gas-phase reactions, another experiment was conducted by exposing a TiO_2_ surface to limonene for 30 min followed by a 30 min evacuation to completely remove limonene in the gas phase, leaving only the TiO_2_ surface covered by adsorbed limonene. HOCl and Cl_2_ were then introduced to the system and were allowed to react with the limonene-TiO_2_ surface for 2 h followed by overnight evacuation. The resulting FTIR spectra (Figure S4) showed similar spectral features to those from Figure 2, suggesting that HOCl and Cl_2_ can react with adsorbed limonene at a surface level without the presence of gas-phase limonene. Most importantly, these surface products remained adsorbed on the surface, even after overnight evacuation.

### Proposed Mechanisms for Identified Products

**Figure 4:**
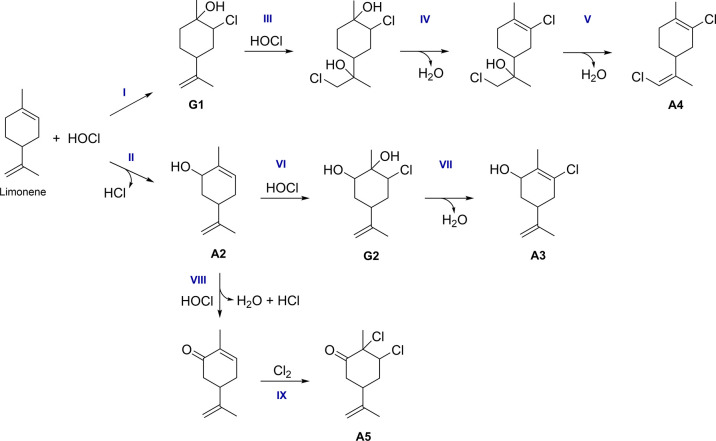
Proposed mechanisms for gas-phase reactions of limonene with HOCl and Cl_2_. The A and G designations are for “adsorbed” and “gas-phase” products, respectively (Table 1).

To understand the chemistry that occurs in indoor environments, several mechanistic pathways (see Figure 4) are proposed based on the identified gas-phase and surface-adsorbed reaction products by HRMS. HOCl is a strong oxidizing agent that readily reacts with unsaturated molecules *via* electrophilic addition across the double bonds to produce chlorohydrins.^25,26^ Dark reactions of limonene with HOCl and Cl_2_ in the gas phase were previously investigated by Wang et al. and found to produce chlorinated limonene.^12^ Most interestingly, it should be noted again that oxygenated/chlorinated surface products may result from adsorption of gas-phase reaction products or from the heterogeneous reactions (i.e., reaction between surface-adsorbed limonene with HOCl/Cl_2_ in the gas phase). Here, we propose a reaction mechanism of HOCl with limonene where HOCl undergoes addition across the double bond of the limonene backbone (**Pathway I**) to form a chlorohydrin (**G1**). Due to the availability of gas-phase HOCl and the remaining exocyclic C=C bond, **G1** may further react with another molecule of HOCl, thereby undergoing another electrophilic addition (**III**) followed by subsequent dehydrations (**IV** and **V**) to form the two C=C bonds in **A4**.

In **Pathway II**, an oxidized limonene product, confirmed to be carveol (**A2**) by HRMS, is generated during the reactions between limonene and HOCl. Therefore, a secondary electrophilic addition (**VI**) can occur, which forms C_10_H_17_O_2_Cl (**G2**) followed by C_10_H_15_OCl (**A3**) after the loss of water (**VII**).

An additional surface product with the addition of two Cl atoms was observed and was attributed to C_10_H_14_OCl_2_ (**A5**). Besides, the FTIR spectra (Figure 2) showed that a carbonyl stretching vibration was present on both the SiO_2_ and TiO_2_ surfaces after exposure to limonene and HOCl/Cl_2_. A loss of the −CO fragment was also observed after MS/MS analysis of *m*/*z* 221.05 (Table 1), confirming the presence of a C=O bond in the **A5** structure. HOCl can oxidize an alcohol to a ketone in an aqueous solution.^23,51^ Therefore, it is possible that carveol may first be oxidized by HOCl (**VIII**) to form carvone. In addition to HOCl reactions, Cl_2_ can similarly undergo electrophilic addition to form a dichloroalkane.^25,52^ Due to the presence of Cl_2_ in the gas phase, it can add across an available C=C bond in a mechanistic pathway similar to that of HOCl (**IX**), resulting in the formation of **A5**. Several compounds formed during gas-phase HOCl and limonene reactions were detected on the SiO_2_ and TiO_2_ surfaces. Similarly, surface-mediated reactions can occur between limonene in the adsorbed phase with gas-phase HOCl and Cl_2_ as discussed earlier. The calculated Δ*G*° values of all proposed reaction pathways are reported in Table S2, confirming that these reactions have negative free energies.

In summary, the application of cleaning products is considered a daily routine for many people around the world. Its significance has become more apparent ever since the COVID-19 pandemic started. Limonene is one of the most abundant indoor VOCs generated from household cleaning products and other sources, such as air fresheners and wood products.^18,53^ Oxidative species emitted from cleaning products, including but not limited to HOCl and Cl_2_, also react with various indoor VOCs emitted from these sources. Moreover, the use of two or more cleaning products simultaneously can lead to the formation of unwanted chemical compounds despite their original purpose: cleaning of surfaces. Our experimental results suggest that the use of chlorine- and terpene-containing household cleaning products leads to formation of less volatile compounds. These compounds not only suspend in the gas phase^11,12^ but also adsorb onto indoor surfaces, effectively increasing their residence times and potentially participating in additional surface chemical reactions. We reveal that SiO_2_ and TiO_2_ are irreversibly adsorbed by gas-phase products between HOCl/Cl_2_ and limonene, including C_10_H_16_O, C_10_H_15_OCl, C_10_H_14_Cl_2_, and C_10_H_14_OCl_2_, which continue to interact with surfaces regardless of evacuation. This result shows that in contrast to our previous study of SiO_2_ with only limonene, where the interaction is reversible,^31,41^ these chlorination and oxidation products exhibit irreversible adsorption. Most interestingly, heterogeneous reactions can occur between surface-adsorbed limonene and gas-phase HOCl/Cl_2_ in which the resulting products adsorb to the surface with minimal desorption for days. Lower-ventilated spaces usually experience higher indoor volatile compound concentrations and residence times, thus providing better landscape for heterogeneous reactions to occur. The concentrations of HOCl, Cl_2_, and limonene used in our experiments are related to the cleaning activities in such low-ventilated indoor settings. Overall, this study shows that surfaces provide a sink for these chlorinated and oxidized compounds. However, over time, the slow desorption of these surface-bound species affects indoor air quality and provides an additional yet overlooked source for human exposure. Further studies conducted on longer time scales, under different environmental conditions, and on potential health implications are warranted.

## Supporting Information Available

Schematic of electrophilic additions of HOCl and Cl_2_ to an unsaturated organic compound; GC-MS data for limonene, mass spectra of four standard compounds (carvone, carveol, alpha-terpineol, and terpinen-4-ol); mass spectra of SiO_2_ and TiO_2_ surface products from FTIR experiments; FTIR spectra of TiO_2_ with consecutive exposures to limonene and HOCl/Cl_2_; table of HRMS isotopic ions; and table of calculated (Δ*G*°) values of all reaction pathways (PDF)
