# Supplementary material for: Gas-Phase and Surface-Initiated Reactions of Household Bleach and Terpene-Containing Cleaning Products Yield Chlorination and Oxidation Products Adsorbed onto Indoor Relevant Surfaces
Source: Environ Sci Technol. 2023 Nov 27;57(49):20699–707. doi: 10.1021/acs.est.3c06656 (PMC10720375; doi:10.1021/acs.est.3c06656)
Supplement: Supplementary file 1 — es3c06656_si_001.pdf [file es3c06656_si_001.pdf]

Supporting information for:

Gas-Phase and Surface-Initiated Reactions of Household Bleach and Terpene-Containing Cleaning Products Yield Chlorination and Oxidation Products Adsorbed onto Indoor Relevant Surfaces

Cholaphan Deeleepojananan[1] *and Vicki H. Grassian*[1*]

[1]Department of Chemistry and Biochemistry, University of California San Diego, La Jolla, CA 92093 USA

*Corresponding Author: Vicki H. Grassian (vhgrassian@ucsd.edu)

Supporting information contains one scheme, four figures and two tables.

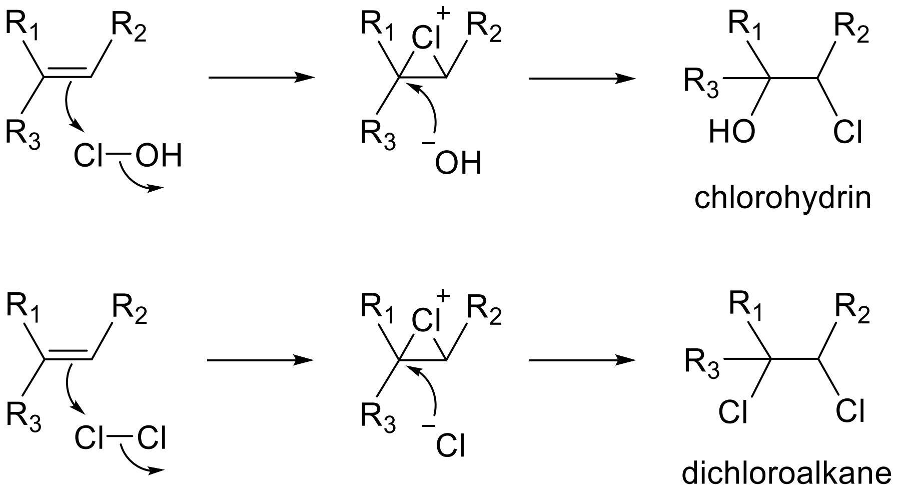

Scheme S1. General electrophilic addition reactions of an unsaturated organic compound with HOCl and Cl_2.[1–3]

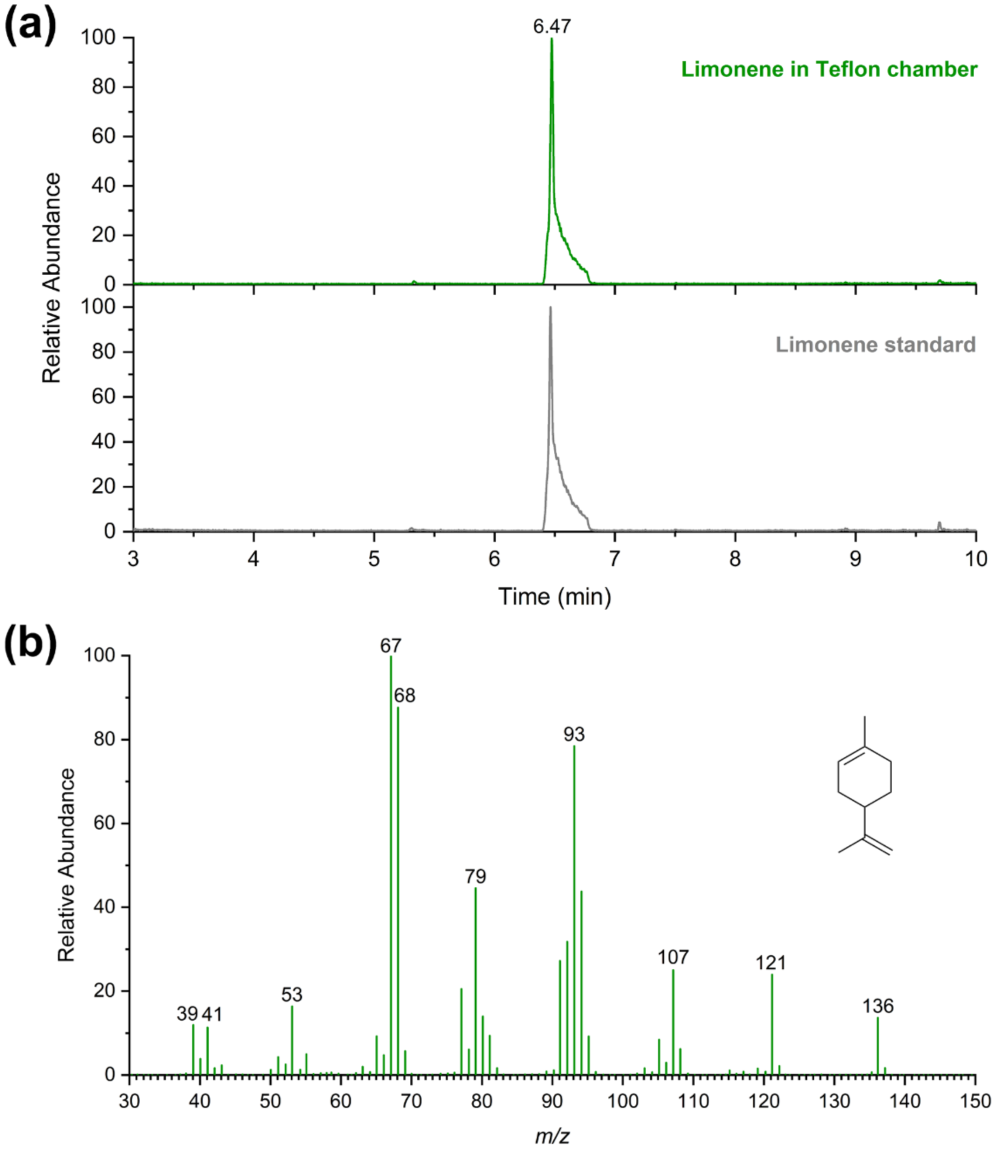

Figure S1. (a) Gas chromatograms of extracted gas-phase limonene in the Teflon chamber compared with limonene standard and (b) corresponding mass spectrum at t = 6.47 min, confirming a limonene structure.

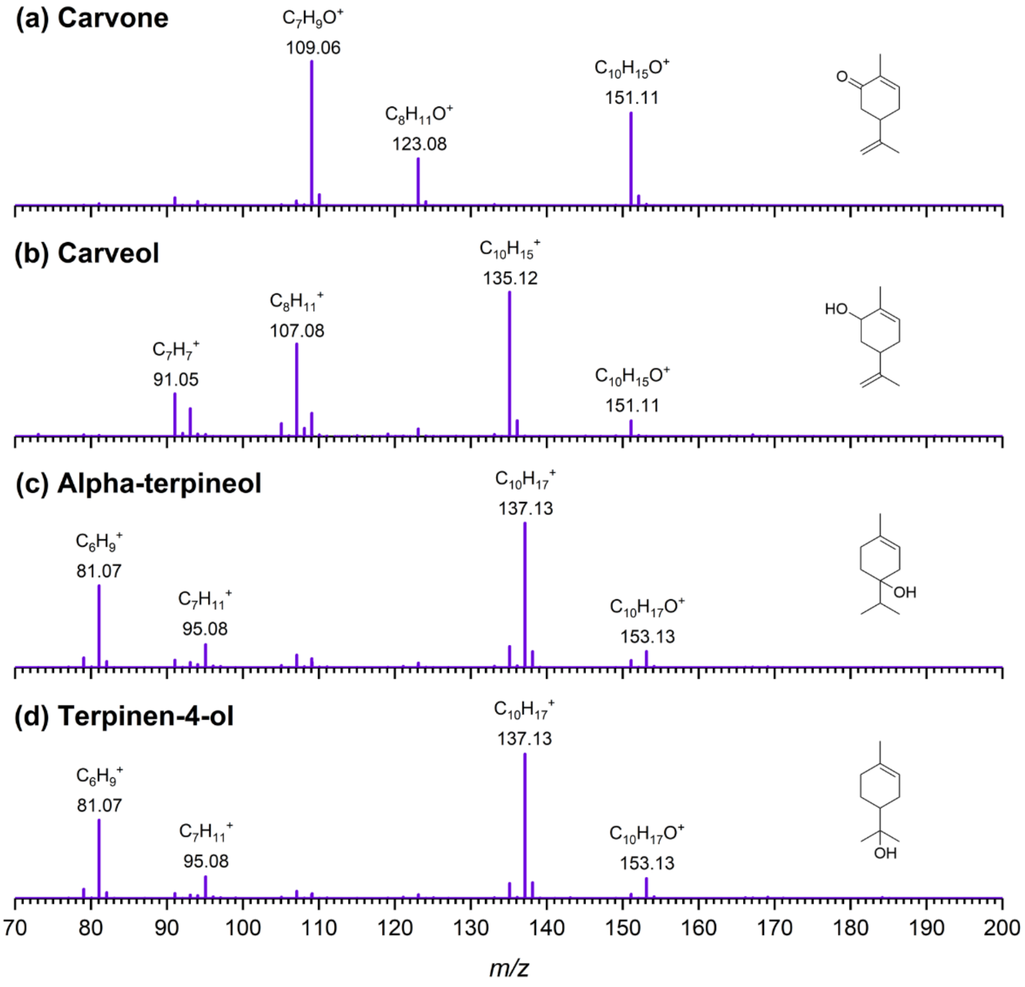

Figure S2. Normalized positive-ion mode mass spectra of 1000 ppm solutions of (a) carvone ($C_{10}H_{14}O$), (b) carveol ($C_{10}H_{16}O$), (c) alpha-terpineol ($C_{10}H_{18}O$), and (d) terpinen-4-ol.

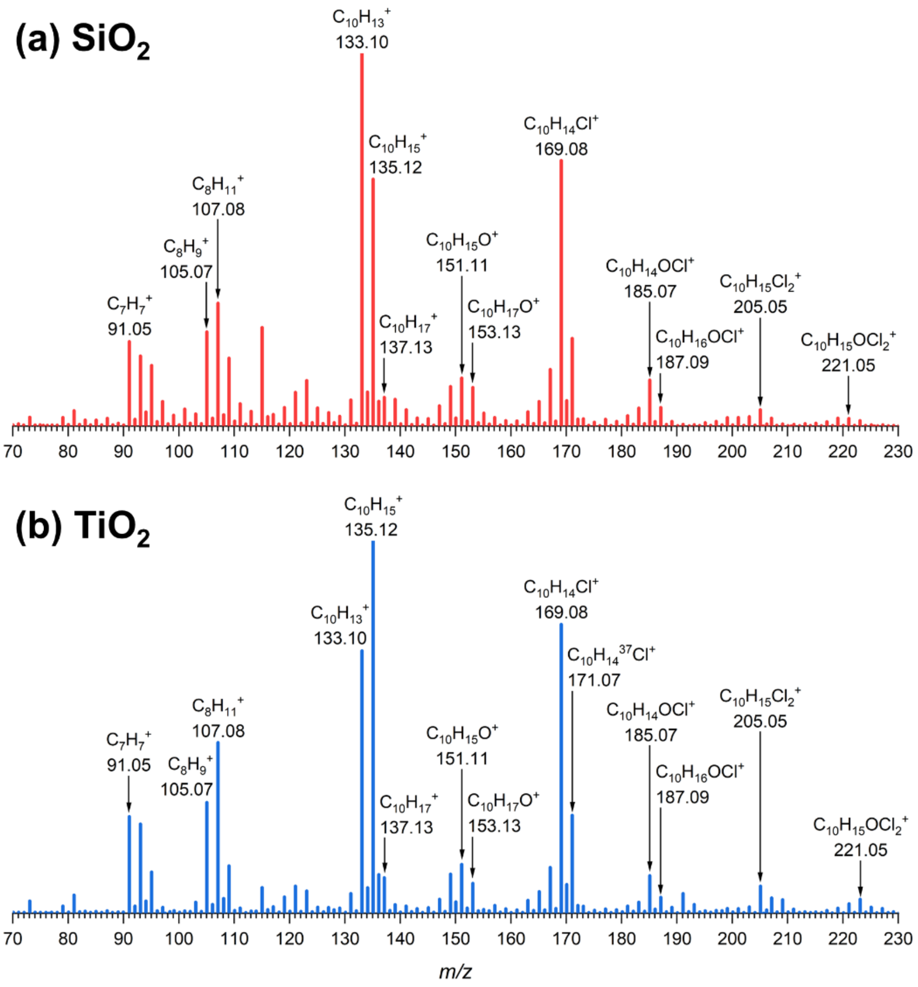

Figure S3. Normalized mass spectra in positive-ion mode of surface products extracted from (a) SiO_2 and (b) TiO_2 after exposure to limonene and $HOCl/Cl_2$ for 2 hours in the t-FTIR cell followed by 1 hour of evacuation.

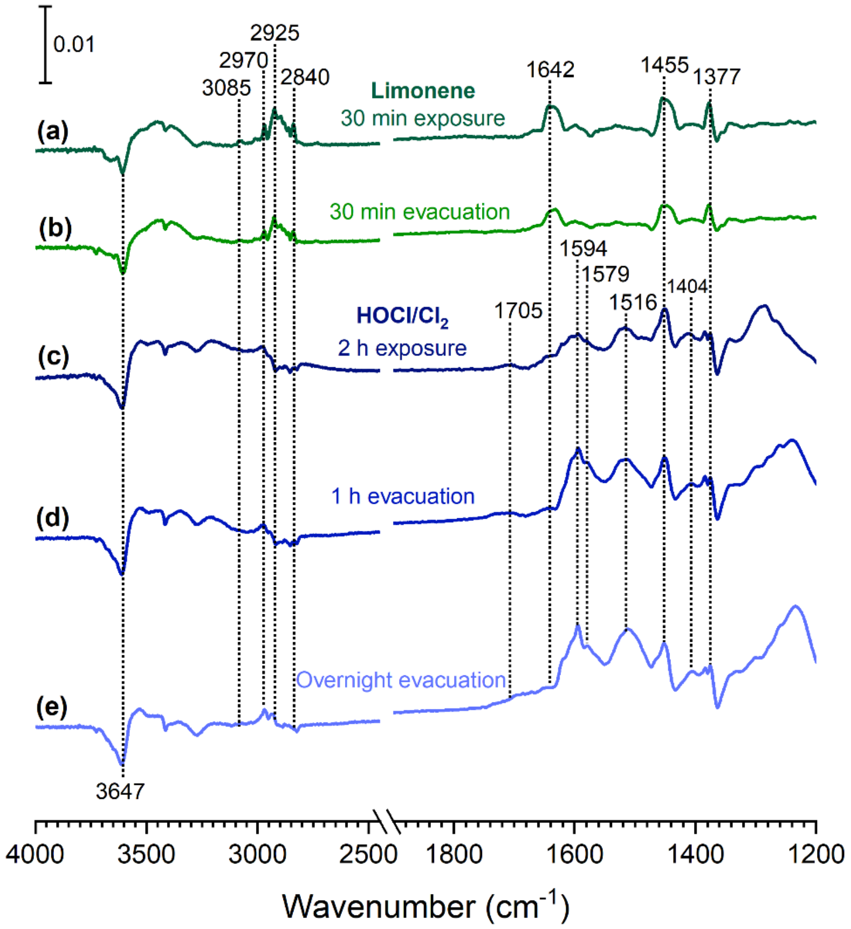

Figure S4. FTIR spectra of TiO_2 (a) after a 30-minute exposure to limonene at an equilibrium pressure of 68 mTorr followed by (b) a 30-minute evacuation. FTIR spectra were subsequently collected after the resulting limonene-TiO_2 was exposed to (c) HOCl and Cl_2 for 2 hours followed by (d) a 1-hour evacuation and (e) overnight evacuation.

Table S1. List of isotopic ions detected in HRMS spectra (**Figure 1** and **Figure S3**)

**Parent *m/z***	**Assigned formula**	**Corresponding isotopic *m/z***	**Assigned formula**
169.08	$C_{10}H_{14}Cl^+$	171.07	$C_{10}H_{14}{}^{37}Cl^+$
185.07	$C_{10}H_{14}OCl^+$	187.07	$C_{10}H_{14}O^{37}Cl^+$
205.05	$C_{10}H_{15}Cl_2^+$	207.05	$C_{10}H_{15}Cl^{37}Cl^+$
		209.05	$C_{10}H_{15}{}^{37}Cl_2^+$
211.09	$C_{10}H_{17}OClNa^+$	213.09	$C_{10}H_{17}O^{37}ClNa^+$
221.05	$C_{10}H_{15}OCl_2^+$	223.05	$C_{10}H_{15}OCl^{37}Cl^+$
227.08	$C_{10}H_{17}O_2ClNa^+$	229.08	$C_{10}H_{17}O_2{}^{37}ClNa^+$

Table S2. Calculated ΔG° values for all proposed mechanism pathways shown in **Figure 4** of the main text.

**Reaction pathway**	**Calculated ΔG° (kJ/mol)**
**I**	-172.1
**II**	-181.2
**III**	-148.7
**IV**	-49.3
**V**	-12.3
**VI**	-159.0
**VII**	-30.7
**VIII**	-279.5
**IX**	-80.4

REFERENCES

(1) Schwartz-Narbonne, H.; Wang, C.; Zhou, S.; Abbatt, J. P. D.; Faust, J. Heterogeneous Chlorination of Squalene and Oleic Acid. *Environ. Sci. Technol.* **2019**, *53* (3), 1217–1224. https://doi.org/10.1021/acs.est.8b04248.

(2) Wang, C.; Collins, D. B.; Abbatt, J. P. D. Indoor Illumination of Terpenes and Bleach Emissions Leads to Particle Formation and Growth. *Environ. Sci. Technol.* **2019**, *53* (20), 11792–11800. https://doi.org/10.1021/acs.est.9b04261.

(3) Spickett, C. M.; Jerlich, A.; Panasenko, O. M.; Arnhold, J.; Pitt, A. R.; Stelmaszyńska, T.; Schaur, R. J. The Reactions of Hypochlorous Acid, the Reactive Oxygen Species Produced by Myeloperoxidase, with Lipids. *Acta Biochim. Pol.* **2000**, *47* (4), 889–899. https://doi.org/10.18388/abp.2000_3944.
